# Driving Institutional Humanization Through Collaborative Governance: A Technical Report From a Portuguese Primary Care Unit

**DOI:** 10.7759/cureus.111417

**Published:** 2026-06-24

**Authors:** Nuno M Nunes, Diogo Martins

**Affiliations:** 1 Family Medicine, Unidade de Saúde Familiar (USF) Gago Coutinho, Unidade Local de Saúde (ULS) do Estuário do Tejo, Alverca do Ribatejo, PRT; 2 Palliative Medicine, Equipa de Cuidados Continuados Integrados (ECCI), Local de Saúde (ULS) do Estuário do Tejo, Vila Franca de Xira, PRT

**Keywords:** administrative burden, clinician well-being, collaborative governance, health policy and management, humanization of care, primary healthcare

## Abstract

The humanization of patient care depends not only on the attitudes and behaviors of individual professionals but also on the organizational conditions in which healthcare professionals work. Following the 2024 reform of the Portuguese National Health Service (Serviço Nacional de Saúde, SNS) and the creation of Local Health Units (Unidades Locais de Saúde, ULSs), primary care organizations were incorporated into more integrated and decentralized governance structures. This technical report describes the implementation of a collaborative governance model between ULS Estuário do Tejo and Family Health Unit (Unidade de Saúde Familiar, USF) Gago Coutinho during the 2025 institutional incentive cycle. The model was designed to translate institutional humanization into practical organizational mechanisms by combining shared priority setting, local needs assessment, centralized procurement support, and investments in professional development, workplace conditions, and team cohesion. The process enabled the unit to fully utilize its available institutional incentive allocation, transforming allocated resources into concrete interventions, including specialized training in family-centered care for autism spectrum disorder (ASD), infrastructure and ergonomic improvements, and a team-building initiative incorporating a community donation component. This experience suggests that institutional incentives can function as health policy instruments when supported by collaborative governance and administrative decoupling. Although this is a single local implementation report, it provides a practical framework for primary care organizations seeking to align professional well-being, organizational performance, and humanized care.

## Introduction

Humanization of care is a central objective of contemporary healthcare systems. Although it is often discussed in relation to patient experience, communication, dignity, empathy, and respect, humanized care also depends on the institutional environment in which professionals deliver care [1]. Healthcare professionals are more likely to provide compassionate, safe, and person-centered care when organizations support their well-being, reduce unnecessary administrative burdens, and create conditions for collaboration, psychological safety, and professional development.

This institutional dimension is particularly relevant in primary care, where multidisciplinary teams are required to respond to complex population needs, chronic disease management, preventive care, family-centered interventions, and community health priorities. In this context, the humanization of care should be understood not only as an interpersonal value but also as an organizational and policy objective. Institutions that invest in professionals, streamline administrative processes, and establish participatory governance mechanisms may be better positioned to deliver patient-centered care.

The Portuguese National Health Service (Serviço Nacional de Saúde, SNS) underwent a major reform in 2024 with the creation of Local Health Units (Unidades Locais de Saúde, ULSs), designed to promote vertical integration, decentralization, and coordination across levels of care [2]. This reform created new opportunities for local leadership structures to align organizational priorities with the needs of specific communities and clinical teams. Within this framework, institutional incentives can serve not only as financial mechanisms but also as instruments of organizational transformation when linked to shared priorities, transparent decision-making, and concrete improvements in professional and patient care environments [3].

The evolution from the Triple Aim to the Quadruple and Quintuple Aim further reinforces the importance of integrating patient experience, population health, cost-effectiveness, clinician well-being, and health equity into healthcare improvement strategies [4]. For primary care units, this requires more than individual motivation. Recent evidence on primary care workforce development highlights the importance of supportive supervision, professional development, teamwork, incentives, and appropriate working environments [5]. At the same time, administrative and documentation burdens have increasingly been recognized as contributors to professional dissatisfaction and burnout, particularly when clinical teams are required to absorb tasks that could be managed through better organizational design and administrative support [6,7].

Healthcare worker well-being has therefore become a major determinant of health system sustainability. Systematic reviews suggest that workplace and organizational interventions can improve well-being, engagement, resilience, and burnout outcomes among healthcare professionals, particularly when they involve concrete changes in work organization, job design, scheduling, or the physical work environment [8,9]. Burnout is also associated with lower quality and safety of care, reinforcing the need to address professional well-being as a system-level responsibility rather than solely an individual issue [10].

This technical report describes the implementation of a collaborative governance model between ULS Estuário do Tejo and Family Health Unit (Unidade de Saúde Familiar, USF) Gago Coutinho during the 2025 institutional incentive cycle. The report focuses on the operational mechanisms used to translate institutional humanization into practical action, including shared priority setting, administrative decoupling, centralized procurement support, and targeted investments in professional training, workplace conditions, and team cohesion.

## Technical report

Setting and institutional context

USF Gago Coutinho is a primary care unit operating within ULS Estuário do Tejo, part of the Portuguese National Health Service (Serviço Nacional de Saúde, SNS). In 2025, the ULS partnered with its USFs to align clinical priorities, organizational governance, and institutional incentives. This approach went beyond conventional administrative supervision to create a partnership among local leadership, clinical teams, and administrative departments.

The central objective of the collaborative governance model was to ensure that institutional incentives were translated into concrete improvements rather than remaining unused because of administrative delays, fragmented decision-making, or unclear responsibilities. The model was based on three operational principles: collaborative governance, administrative decoupling, and holistic investment.

Collaborative governance refers to the development of a continuous partnership among the Board of Directors, the Clinical Direction of Primary Healthcare, the ULS procurement structures, and the USFs. Administrative decoupling refers to the redistribution of procurement and logistical responsibilities away from clinical professionals and toward administrative departments with appropriate technical expertise. Holistic investment refers to the use of incentives not only for clinical tools and equipment but also for professional development, workplace conditions, team cohesion, and community engagement.

Operational framework: collaborative governance model

**Figure 1:**
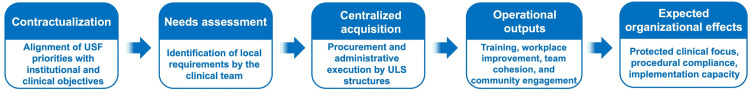
Collaborative governance model for institutional incentive execution. The model combines contractualization, local needs assessment, and centralized acquisition to translate institutional incentives into implemented interventions while reducing administrative burden on clinical professionals and preserving fiscal and procedural compliance. ULS: local health unit, USF: family health unit. Note: This image was created by Diogo Martins using Microsoft PowerPoint (Microsoft Corporation, Redmond, WA, USA). No artificial intelligence assistance was used.

The implementation process followed a three-stage lifecycle: contractualization, needs assessment, and centralized acquisition (Figure 1). The first stage was contractualization. During this phase, the priority areas of the USF were identified and aligned with the institutional and clinical objectives established for the incentive cycle. This ensured that the use of incentives was linked to recognized organizational needs rather than to isolated or opportunistic purchases.

The second stage was needs assessment. The USF identified specific requirements that could support clinical activity, professional well-being, and organizational culture. This process involved translating broad strategic priorities into practical interventions. The unit prioritized investments that could support vulnerable populations, improve working conditions, and strengthen team functioning.

The third stage was centralized acquisition. After the identification of needs and the collection of relevant procurement information, the Clinical Direction of Primary Healthcare and the ULS procurement department assumed responsibility for the administrative and purchasing processes. This step was essential because it reduced the administrative burden placed on healthcare professionals and allowed clinical teams to remain focused on patient care while also ensuring fiscal and procedural compliance.

This operational structure created a clear division of responsibilities: the clinical team identified needs and priorities based on local knowledge; leadership structures validated the strategic alignment of proposed investments; and the procurement department executed the administrative process. This division supported both accountability and efficiency.

Implementation at USF Gago Coutinho

Within this framework, USF Gago Coutinho achieved full execution of the available institutional incentive allocation during the 2025 cycle. In this report, full execution refers to the implementation of all approved initiatives funded through the available institutional incentive allocation. This 100% execution rate represented an operational benchmark because allocated resources were translated into implemented interventions. In many healthcare settings, institutional funds may remain partially unused because of procurement complexity, time constraints, lack of coordination, or limited administrative support. In this case, the governance model enabled the unit to move successfully from planning to execution.

The unit’s implementation strategy was based on three practical pillars: planning, involvement, and commitment. Planning consisted of identifying needs early in the incentive cycle and aligning them with clinical and organizational objectives. Involvement consisted of engaging the team in decisions regarding the use of incentives, thereby increasing ownership and transparency. Commitment consisted of maintaining a shared focus on moving approved initiatives from proposal to implementation.

The implemented portfolio included three approved intervention areas: autism-focused professional training; workplace infrastructure and ergonomic improvements; and a structured team-building initiative with a community donation component. Table 1 summarizes the implemented interventions, their organizational rationale, their contribution to institutional humanization, and suggested indicators for future evaluation.

**Table 1:** Institutional incentive portfolio and evaluation indicators. ASD: autism spectrum disorder, ULS: local health unit, USF: family health unit.

Investment area	Implemented intervention	Organizational rationale	Supported dimension of institutional humanization	Suggested indicators for future evaluation
Professional training	Family-centered ASD care training	Improve professional knowledge and confidence in caring for autistic patients and families	Specialized, person-centered and family-centered care	Training participation, self-efficacy, professional confidence, patient/family feedback
Workplace conditions	Infrastructure and ergonomic improvements	Improve daily working conditions and signal institutional attention to staff needs	Professional well-being and workplace humanization	Staff satisfaction, perceived ergonomic adequacy, absenteeism, burnout indicators
Team cohesion	"Bike Building" team initiative with community donation	Strengthen communication, trust, shared identity, and community engagement	Team functioning, psychological safety, and social responsibility	Team climate, psychological safety, staff engagement, community partnership outputs

Specialized professional training focused on family-centered care for patients with autism spectrum disorder (ASD). This training addressed the need to improve the unit’s capacity to respond to patients and families with specific neurodevelopmental and social needs. This investment is supported by evidence showing that healthcare professionals often report limited ASD-specific knowledge and self-efficacy and that specialized ASD-related training programs can improve physicians’ knowledge and confidence in caring for autistic patients [11,12]. The strategic relevance of this intervention lies in extending humanization beyond general communication principles and applying it to a vulnerable population requiring tailored, family-centered approaches.

A second investment area focused on infrastructure and ergonomics. Incentives were used to acquire equipment aimed at improving daily working conditions for professionals. This intervention reflected the principle that workplace humanization is a necessary component of care humanization. By improving the physical and functional environment in which professionals work, the unit sought to support professional well-being, reduce avoidable strain, and reinforce institutional attention to staff needs.

A third intervention focused on team cohesion through a structured team-building initiative. The “Bike Building” activity required team members to collaborate on challenges, accumulate points, and convert those points into components used to assemble four bicycles. The activity had both internal and external objectives. Internally, it was designed to strengthen communication, collaboration, and a sense of belonging within the team. Externally, the completed bicycles were donated to a local institution, linking team development with community engagement. In the context of primary care, where interprofessional collaboration has been associated with improved management of several patient groups, effective team functioning is an important organizational asset [13].

The combination of these interventions allowed institutional incentives to be applied across clinical, organizational, and social dimensions. Rather than concentrating resources on a single type of expenditure, the unit adopted a diversified strategy that integrated professional development, workplace conditions, team functioning, and social responsibility.

## Discussion

The experience of USF Gago Coutinho shows that the practical value of institutional incentives depends less on their availability alone than on the governance conditions that enable their execution. In this case, shared priority setting, administrative support, and centralized procurement created an operational pathway through which clinical priorities could be translated into implemented interventions.

The 100% execution rate achieved by the unit is important as an operational indicator. It demonstrates that allocated resources were effectively translated into implemented actions. However, this indicator should be interpreted with caution. Full financial execution does not, by itself, demonstrate improvements in patient experience, workforce well-being, safety culture, or clinical outcomes. Rather, it indicates that the governance and procurement processes functioned effectively enough to transform planned incentives into concrete investments.

The main contribution of this report is the description of a potentially transferable operational model. First, collaborative governance allowed institutional and clinical actors to work toward shared priorities. Second, administrative decoupling protected clinical professionals from excessive involvement in procurement processes, allowing them to focus on care delivery and local needs identification. Third, holistic investment ensured that incentives were directed not only toward technical or material resources but also toward training, ergonomics, team cohesion, and community engagement.

The model is aligned with broader healthcare improvement frameworks that recognize clinician well-being as a component of system performance [4]. In primary care, where teams face increasing clinical complexity and administrative demands, organizational support is essential. The present case is consistent with recent evidence emphasizing that primary care workforce development requires integrated strategies addressing training, team functioning, working conditions, support structures, and incentives [5]. In this report, the incentive mechanism created a structured opportunity to link workforce development, workplace improvement, and team cohesion within a single implementation cycle.

The policy relevance of administrative decoupling is also supported by recent evidence on administrative and documentation burden in healthcare. Administrative burden in primary care can reduce professional satisfaction and contribute to burnout, while documentation burden has become an important measurable dimension of healthcare work [6,7]. The model described in this report did not remove administrative requirements; rather, it redistributed responsibilities so that procurement and logistical processes were managed by administrative structures with appropriate expertise. This approach preserved transparency and compliance while protecting clinical teams from avoidable non-clinical workload.

The investments in workplace conditions are consistent with the growing literature on organizational interventions for healthcare worker well-being. Workplace interventions have been shown to improve well-being, engagement, resilience, and burnout-related outcomes among healthcare professionals [8]. More specifically, organizational interventions involving job and task modifications, scheduling changes, and changes to the physical work environment appear particularly relevant for improving mental health and reducing burnout among healthcare workers [9]. Although the present report did not measure burnout directly, this evidence supports the rationale for using institutional incentives to fund workplace-level improvements rather than relying exclusively on individual-level resilience interventions.

The team-building component should be interpreted through the lens of team effectiveness and psychological safety rather than as a recreational activity. Teamwork in healthcare is associated with safer, higher-quality care, and systematic reviews suggest that teamwork is positively associated with clinical performance [14,15]. Interventions to improve healthcare team effectiveness include team training, tools, organizational redesign, and structured programs [16], while evidence-based frameworks emphasize accountability, conflict management, decision-making, reflection, and coaching as common targets for improving teamwork [17]. In this report, the Bike Building initiative is best understood as a structured intervention intended to strengthen communication, trust, shared identity, and community engagement, which are plausible antecedents of safer, more coordinated, and more socially connected care.

Psychological safety remains particularly relevant in this context. Edmondson’s foundational work defined psychological safety as a condition that enables learning behavior within teams [18]. Subsequent healthcare literature has identified factors that promote psychological safety in healthcare teams, including leadership support, familiarity, status management, and inclusive communication [19]. Recent evidence suggests that psychological safety may be associated with patient safety outcomes, although the evidence remains heterogeneous and requires further empirical clarification [20]. For this reason, the present report adopts cautious language: the initiative may support conditions associated with safer teamwork, but it does not demonstrate improvements in patient safety without direct measurement.

Several limitations must be acknowledged. First, this is a single-unit technical report and does not include a comparator group. Second, the report describes implementation and operational execution but does not provide pre- and post-intervention measurements of professional satisfaction, burnout, psychological safety, patient experience, access, or clinical outcomes. Third, the reported interventions occurred within a specific institutional and legal context, which may limit direct transferability to other health systems. Fourth, the 100% execution benchmark reflects implementation success but should not be interpreted as evidence of clinical effectiveness. Future evaluations should include quantitative and qualitative indicators, including staff experience, procurement timelines, patient-reported experience measures, and selected quality and safety indicators.

Despite these limitations, the experience provides practical lessons for primary care governance. Institutional humanization requires more than values-based language. It requires operational pathways through which leadership, administration, and clinical teams can jointly identify priorities, mobilize resources, and implement improvements. When incentives are supported by collaborative governance, they may become instruments for strengthening organizational resilience, professional engagement, and patient-centered care.

## Conclusions

The experience of USF Gago Coutinho and ULS Estuário do Tejo suggests that institutional incentives can support healthcare humanization when embedded within a collaborative governance model. The combination of shared priority setting, administrative decoupling, centralized procurement support, and holistic investment enabled the full execution of the available institutional incentive allocation and supported interventions targeting professional training, workplace conditions, team cohesion, and community engagement.

This report provides a practical framework for primary care organizations seeking to translate institutional humanization from a normative principle into operational practice. Future evaluations should assess whether this model is associated with measurable improvements in professional experience, patient experience, procurement efficiency, quality of care, and organizational sustainability.
